# Past Is Prologue: Dismantling Colonial Legacies to Advance Black Health Equity in the United States

**DOI:** 10.1089/heq.2023.0226

**Published:** 2023-12-12

**Authors:** Sirry M. Alang, Chelsey R. Carter, Oni Blackstock

**Affiliations:** ^1^ Department of Health and Human Development, University of Pittsburgh, Pittsburgh, Pennsylvania, USA.; ^2^ Department of Social and Behavioral Sciences, Yale School of Public Health, New Haven, Connecticut, USA.; ^3^ Health Justice, New York, New York, USA.

**Keywords:** colonialism and health, decolonizing medicine, anti-racism in medicine, health equity for Black Americans

## Abstract

Colonialism underlies the commodification of health care in the United States and continues to harm well-being among Black Americans. We present four recommendations for addressing its health consequences: (1) Investments in epigenetic research to improve our understanding of how systemic oppression becomes biology. (2) Centering Black experiences and knowledge traditions in education, practice, and policy. (3) Support for Black scholars, trainees, and practitioners when they critic disciplinary tenets and practices. (4) Expansion of preventive care. Our health care system is a for-profit industry that exploits workers and harms the most marginalized, much like colonialism. Advancing health equity requires dismantling colonial legacies.

On September 8, 2022, the British Empire's longest serving monarch, Queen Elizabeth II, died. Her death generated conversations about the role of British colonialism and slavery in the suffering of people in territories seized by the British Empire.^1^ Colonialism is not in the distant past and its health impacts are enduring.^2^ As Black women with diasporic ancestries in the Caribbean and Africa, we understand firsthand that personal narratives and collective histories of colonial subjects are not abstract theoretical debates. Instead, collective histories and experiences offer a much needed sociocultural and historical perspective to advance understandings of how structural inequalities produce inequitable health outcomes, and how to address them.^3,4^

Our essay makes visible the ways by which colonialism harms the health of Black communities in the United States. While colonialism has significantly affected many ethnoracial groups globally, we focus on Black Americans who are not often seen as direct victims of British colonialism. Given that chattel slavery in the United States was an expansive colonial project, this analysis considers the mechanisms that connect colonialism to negative health outcomes among Black Americans and provides recommendations for scholars, policymakers, and practitioners.

Broadly, colonialism is the subjugation of communities by external and powerful groups. In settler colonialism, Indigenous populations are displaced by colonizers who stay to form permanent communities.^5^ In the Americas, former British colonies that later became the United States were formed by genocide of Indigenous Peoples. European colonizers seized lands and resources of nations that inhabited the lands centuries before European invasion, all while kidnapping and enslaving human beings from Africa to these new colonies.^5^

The transatlantic slave trade was the most pernicious and enduringly exploitative strategy to advance colonial projects that sought to expand European capitalist interests and domination in the Americas.^5^ The slave trade through the Royal African Company was the biggest economic enterprise in the 18th century, and it was dominated by the British Empire.^6^ Stolen Africans were murdered, and subjected to brutal and unsanitary conditions that caused suicide, sickness, injury, disability, and death.^7–9^ This violent legacy did not only exist during the Middle Passage to the Americas but was bolstered after American independence and sustained even after Emancipation.^5^

## Colonialism and Health

Health and health care experiences of Black Americans are directly linked to settler colonialism and chattel slavery. Often, scholars engage with these systems as if they remain in the past. However, processes such as mass incarceration and racial residential segregation that impact health are also colonial projects to advance racial capitalist expansion. Colonialism necessitates subjugation, forced labor, displacement of people who are not white, and exploitation of resources.^5,10^ Mass incarceration operates in similar ways. It decimates Black families, and the prison industry profits from the exploitation of disproportionately incarcerated Black Americans. Indeed, incarceration and segregation sustain exploitation and unequal distribution of resources that benevolently follow White Americans over the life course and across generations while simultaneously oppressing Black communities.^5,8,10^ These colonial legacies impact the health of Black populations in the United States.

In what follows, we detail four ways by which colonialism harms health in Black communities:

1. *Intergenerational transmission of trauma and embodied inequality: C*olonialism and chattel slavery constitute traumatic histories of Black communities that become embodied. Transgenerational trauma—the consequences of trauma that span across generations—harms health.^11^ While race is a social construct that does not mark racialized persons as biologically different from white bodies, the traumatic impacts of racism become biology.^12^ Biological memories of traumatic experiences are embedded in the human genome, transmitted across generations, and contribute to racial inequities in health.^11^ Inequalities in social and environmental conditions, and psychosocial stressors of racism can alter gene expressions in ways that increase vulnerability to disease.^11^ Therefore, ancestral experiences of colonialism and slavery and exposure to its contemporary forms are traumas relevant to the medical histories and health of Black patients.
2. *Eurocentrism in medicine:* Medical education and health care delivery continue to be based on white norms and values, ignoring other forms of knowledge.^13^ Evidence that shapes the practice of medicine is overwhelmingly generated from research that often excludes Black communities—their experiences, histories, and perspectives,^14^ or are based on experiments that exploited and harmed them.^15^ Academic medicine is also deficient of Black critically trained scholars who could apply antiracist and anticolonial frameworks in teaching and clinical practice.^16^ The systematic exclusion of these scholars in academic medicine and in managerial positions within health systems is detrimental to the health of all communities, but especially Black communities because it ignores their needs and perspectives and likely perpetrates policies that harm them.
3. *Ongoing influences on the social determinants of health*: Colonialism shapes the social determinants of health. For example, racial residential segregation, neighborhood-level economic inequities, and police brutality are colonial legacies. Modern-day policing evolved from organized groups of armed white men who monitored enslaved and freed Black people—whipping, arresting, and lynching them to enforce Jim Crow laws, ensure captivity, and maintain white dominance over resources.^17^ In addition to causing morbidity and mortality, colonial legacies harm health indirectly by limiting access to financial, social, and economic resources.^2^ Colonialism and the slave trade have also shaped how money and power are distributed locally, nationally, and globally^5^—impacting neighborhood and work conditions, housing, education, and other social determinants of health.^2^
4. *The commodification of health care*: In the United States, medical care is often treated as a profit-making entity rather than investments in well-being.^18^ The health care industry, like colonialism, is driven by capitalism. Commodification has led to the prioritization of patients with resources who are disproportionately white. Resources, consumer ability, and choice are prioritized over equity and access.^18^ Powerful interest groups continue to benefit from the capitalism that defines the health care industry at the expense of racially minoritized commuinties.^19^ This limits access to quality care and prevents the establishment of safety-net programs that improve well-being of those with limited resources.^19,20^

## Addressing the Impacts of Colonialism on Health

Colonialism is maintained by multiple institutions and policies such as incarceration, housing, education, and medicine.^5,9,10,13^ Addressing its impact on health requires working within and across different systems to implement policies and practices that decenter whiteness—a dominant cultural space that sustains itself by keeping others at the margins.^21^ The following five recommendations, while not exclusive, are specific to the fields of health and medicine.

First, research to help expand our understanding of mechanisms that underlie the intergenerational transmission of racial trauma is important. Available epigenetics scholarship that assesses how inequality shapes gene expression and health focuses on individual characteristics like race as proxies for broader social stratification processes such as racism, colonialism, and slavery.^22^ Exploring these broad structural processes will advance our understanding of how inequality gets under the skin, and might inform the development of interventions to interrupt pathways that connect inequality to illness across generations. Substantial research investments in these areas are necessary.

Second, given the connections between past exposure to inequality and future health, policies that level the playing field like affirmative action and decarceration are paramount for improving future health. Increasing access to high-quality education for Black students, mandating living wage, and breaking the school-to-prison pipeline through evidence-based interventions are also policies that we should advocate for as they redistribute resources between those who benefit from colonialism and those harmed by the same.

Third, recruitment and retention of Black scholars in academic medicine and other health fields are important for providing structurally competent and culturally relevant care. Institutions must be prepared to fully support these scholars when they ask questions that challenge disciplinary, professional, and institutional tenets and practices. Institutions must also value the expertise of Black scholars, especially expertise that reflects their lived experiences. Decentering whiteness requires valuing other forms of knowledge creation such as experiential, indigenous, and critical knowledge processes.

Fourth, education should include lessons about the collective traumatic histories of Black populations in the United States and worldwide. This knowledge makes racism and colonialism visible, facilitates their elimination, and also facilitates the provision of patient-centered care, management of triggers, and identification of resources that communities might need to buffer the impacts of systemic and psychosocial stressors that are linked to colonial legacies.

Finally, we must guarantee access to preventive care. A single payer system, such as Medicare for All, will facilitate access to preventive care especially for communities harmed by slavery and colonialism.^23^ Indeed, the current health insurance system mimics eras when plantation owners and colonial masters prevented enslaved and colonial subjects from receiving care until their conditions became very severe. The focus on profits generated from the health care industry and exploitation of the labor of Black health care workers, are colonial legacies that must end if we seek to advance racial health equity.

## Conclusion

Scholars, practitioners, and policymakers should not ignore the impact of colonialism on the health of Black Americans. We must reinforce our commitments to sustained antiracism in health and medicine. Re-learning and engaging history to actively address the “invisible” yet insidious harms of colonialism on the health of Black people in the United States are important steps toward health equity.
